# Energy big data abnormal cluster detection method based on redundant convolution codec

**DOI:** 10.1038/s41598-024-59373-0

**Published:** 2024-04-15

**Authors:** Rui Ma, Zhenhua Yan, Jia Liu, Wenni Kang, Dongge Zhu

**Affiliations:** Electric Power Research Institute of State Grid Ningxia Electric Power Co., Ltd., Yinchuan, 750002 Ningxia China

**Keywords:** Redundant convolutional codec, Energy big data, Abnormal cluster, Testing, Cluster, Energy science and technology, Engineering

## Abstract

Due to the scattered distribution and poor clustering of abnormal clusters in energy big data, the ability to detect anomalies is poor. Therefore, a high-energy data anomaly clustering detection method based on redundant convolutional encoding is proposed. Quantitative analysis of the coupling characteristics of electrical thermal gas optical time series for multi energy users based on Copula function, and incorporating quantitative values into multi energy feature indicators to extract the energy consumption behavior characteristics of multi energy users. Utilize redundant convolutional codecs to recombine and structurally encode abnormal features of energy big data, and capture multi energy coupling time features using coupling time capsule layers. Then, coupling time features are synthesized through fully connected linear regression layers to generate anomalous clustering feature components, and the energy time series data is then transformed into feature values of the time series in three-dimensional space. Based on this, a comprehensive energy system and massive multi energy user energy big data anomaly clustering analysis are carried out to determine the optimal number of multi energy users. Then, based on linear layers, the electricity heat gas light load characteristic map of multi energy users is transformed into one-dimensional form, and an energy big data anomaly clustering detection model is constructed to complete anomaly detection. The simulation results show that the proposed method has excellent feature clustering performance, detection accuracy above 98.7%, fast convergence speed, and an error rate below 0.1, which has reliable application value.

## Introduction

Big data of energy is an important means to comprehensively collect, process, analyze and apply so as to realize the government's energy supervision, promote the social sharing of energy information. Big data technology has been widely researched and applied in various fields of energy. In the field of energy planning, it has assisted in energy demand forecasting and energy planning research. In the field of energy production^1^, it has been applied to power station operation monitoring, renewable energy generation power forecasting, trans-regional new energy consumption, etc. In the field of energy consumption, research on user-side energy management and parallel load forecasting based on big data has been carried out. At present, there are some problems in the application of energy big data, such as immature circulation and sharing mechanism, relatively narrow data sources, different technical standards, etc. At the same time, the research and application supporting the macro-management level of energy is still relatively weak, which affects the supervision efficiency and scientific decision-making effect of energy industry in China, and leads to the abnormality of energy big data. Therefore, in order to enhance the ability of energy big data information management, it is very necessary to detect anomalies accordingly^2^.

With the promotion of new energy technologies, such as renewable energy, distributed power generation and other advanced technologies like big data, blockchain and artificial intelligence, the energy Internet has further developed^3^. Every node existing in the energy Internet will generate a large amount of data in the process of energy production, transportation, storage, etc. These data will be stored in different regions, different enterprises, or different departments of enterprises, and the data will not be shared with the outside world, which will make the energy data fail to exert its value and hinder its further utilization^4^. In the energy Internet, the sharing of energy data across regions, enterprises and departments can make better use of the value of energy data, which is also conducive to the development and progress of the energy Internet. At present, when access control of energy data is realized within energy companies and energy data is shared among different energy companies, there are some problems in the shared data, such as privacy is easy to leak out, data security cannot be guaranteed, data provided by data providers is not credible, data ownership is lost due to copying and forwarding, etc.^5–7^, which become obstacles to the process of data sharing. Aiming at the problems of data tampering, high risk of leakage and easy loss of data ownership in the process of energy data sharing, access control technology is an important technical means to protect data privacy and security. Attribute encryption technology can perform fine-grained access control on energy data, and the combination of blockchain technology and attribute encryption technology is more conducive to access control of internal data of energy companies and data sharing among energy companies. At present, big data will face many security risks in the process of production, storage and use, and these data often contain a lot of user privacy. When the data is leaked, the user privacy implied in the data will be exposed, which will bring a lot of troubles and troubles to users. Energy data involves the energy data of many enterprises and citizens, as well as the production and sales data of the energy industry. Once there is a problem in data security, there will be very bad consequences. By combining data access control technology with blockchain technology with the characteristics of trustworthiness, tamper resistance, traceability and encrypted transmission, a set of secure, private and mutual trust energy data access control scheme is provided. The correlation analysis of energy time series refers to the quantitative analysis of the coupling characteristics between multi-energy time series according to the historical data of multi-energy time series, so as to obtain the analysis results, and then select the influencing factors of the multi-energy time series prediction data set, which is conducive to improving the accuracy of multi-energy time series prediction. For anomaly detection, relevant scholars have also carried out research in this regard. For example, an anomaly detection method based on neural system recognition and Bayesian filtering was proposed in literature^8^. The neural network architecture is used for system identification, that is, to capture the dynamics of CPS in the dynamic state space model. Bayesian filtering algorithms are then naturally applied on top of the "identified" state-space model to perform robust anomaly detection by recursively tracking the uncertainty of the system's hidden state over time. Reference^9^ proposes an anomaly detection method for supervised deep transfer learning (DTL). The use of 2D image representations allows the utilization of any underlying data in the feature set, providing more possibilities for encoding and detecting relevant features of the data that may not be considered in standard 1D time series. Using the effect of CNN activation as a feature of a machine learning (ML) model, the concept of layer and hyperparameter variation of the CNN model reduces the total time calculation and resource requirements of the proposed system to complete anomaly detection. In literature^10^, micromotion and improved K-nearest neighbor anomaly detection methods were used. Define and assign normal and abnormal power consumption, introducing a rule-based algorithm to extract micromotions that represent intention-rich moments where the end user makes a decision to consume energy; An improved K-nearest neighbor model is introduced to automatically classify the consumption footprint as normal or abnormal to complete the detection. However, the above methods are used in anomaly detection of energy big data. Due to the high dimension of energy big data, the two methods cannot accurately extract important features in practical applications, which affects the accuracy of subsequent detection results.

Aiming at the problems of traditional methods, an anomaly cluster detection method based on redundant convolutional coding for energy big data is proposed to enhance the detection ability of anomaly clusters in energy big data. The coupling-time capsule layer is used to capture the coupling-time characteristics of multi-energy, and the cluster analysis of comprehensive energy system and massive multi-energy users is carried out, and the optimal number of multi-energy users is determined. The coupling-time characteristics are integrated by the fully connected linear regression layer, and the model parameters are adjusted by the double-layer iterative training method to speed up the convergence speed. Finally, the experimental test verifies that the proposed method has high performance and superiority in anomaly detection and recognition, and can provide reliable value reference for energy big data anomaly detection.

## Energy time series and multi-energy characteristic index

### Time series analysis of energy data

Due to the interdependence and coupling characteristics of multiple variables in energy big data, traditional univariate statistical methods may not be able to accurately capture these complex relationships. Copula function is a method used to describe the correlation between multi-dimensional random variables, which can quantitatively analyze the coupling characteristics of electric-heat-gas-optical time series of multi-energy users. By using Copula function, we can quantify the dependence between multiple variables more accurately and extract the behavior characteristics of energy consumption, so as to meet the needs of anomaly clustering detection of large energy data. Thus, based on Copula function, the coupling characteristics of multi-energy users' electricity-heat-gas-light time series are quantitatively analyzed, and the quantified values are included in the multi-energy characteristic index^11^. The collected data are fused by using the cluster structure. Considering the strong coupling characteristics among multi-energy loads in the energy system and the randomness and variability of time series, the prediction accuracy of multi-energy time series is affected, and the envelope amplitude distribution of energy data time series is given as follows:

1 $$ x_{n} = a_{0} + \sum\limits_{i = 1}^{{M_{AR} }} {a_{i} x_{n - i} + } \sum\limits_{j = 0}^{{M_{MA} }} {b_{j} } \eta_{n - j} , $$

wherein, $a_{0}$ is the distribution of electrical load, thermal load, gas load and photovoltaic output nodes, $x_{n - i}$ is the probability density at any time point, $b_{j}$ is that each node has the corresponding multi-modal characteristic distribution, $\eta_{n - j}$ is the mean of the envelope distribution at the $n - 1$ th point in time, $a_{i}$ is the observed value of the $i$ th autoregressive coefficient in the time series, $M_{AR}$ is the autoregressive coefficient, and $M_{MA}$ is the moving average coefficient.

The parameter estimation method, which assumes that random variables obey a certain distribution, is not applicable. When fitting the distribution function, the nonparametric method is based on the characteristics of the random variable sample point set itself^12^, without determining the specific distribution of random variables in advance, so it has strong objectivity. According to the neighbor set of node I, if the window width W is too large, it is difficult to capture some important features in the edge distribution of multi-energy time series, resulting in large deviation in the estimation of the edge distribution. The energy data anomaly detection function is given as follows:

2 $$ \mathop {\max }\limits_{{x_{a,b,d,p} }} \sum\limits_{{a \in {\text{A}}}} {\sum\limits_{{b \in {\text{B}}}} {\sum\limits_{{d \in {\text{D}}}} {\sum\limits_{{p \in {\text{P}}}} {x_{a,b,d,p} V_{p} } } } } $$

3 $$ {\text{s}}.{\text{t}}. \sum\limits_{{a \in {\text{A}}}} {\sum\limits_{{d \in {\text{D}}}} {\sum\limits_{{p \in {\text{P}}}} {x_{a,b,d,p} R_{p}^{bw} \le } } } K_{b}^{bw} \left( {\text{S}} \right),b \in {\text{B,}} $$

wherein, $x_{a,b,d,p}$ is the M-th electric load time series and gas load time series, $V_{p}$ is the integrated moving average autoregressive parameter, $R_{p}^{bw}$ is the joint multiplier of real power generation and minimum production, $K_{b}^{bw} \left( {\text{S}} \right)$ is the fuzzy membership degree of energy big data in new energy generation mode, ${\text{A}}$ is the original observed value of the energy data, ${\text{B}}$ is the final category of abnormal data, ${\text{D}}$ is abnormal score, and ${\text{P}}$ is the probability of anomalies.

In order to analyze the correlation between different energy flow time series of users more intuitively, it is necessary to use other evaluation indexes to describe the correlation characteristics between multi-energy time series. At present, the proposed metrics to describe the correlation between multivariate random variables mainly include Pearson correlation coefficient, Kendall rank correlation coefficient and Spearman rank correlation coefficient. Among them, Perason correlation coefficient is the most common linear correlation coefficient, which is used to analyze the degree of linear correlation between multivariate random variables. However, there is a nonlinear correlation between the time series data of electricity-heat-gas-light of multi-energy users. However, there is a nonlinear correlation between the data of multi-energy users' electricity-heat-gas-light time series, so Pearson relation number is not suitable for the correlation analysis of multi-energy users' electricity-heat-gas-light time series in integrated energy system^13^. In this section, Spearman rank correlation coefficient is selected to measure the nonlinear correlation between electricity-heat-gas-light time series data of multi-energy users in integrated energy system^14^.

### Quantitative analysis of multiple energy characteristics indicators

And its quantitative value is included in the multi-energy characteristic index, so as to extract the energy consumption behavior characteristics of multi-energy users. Redundant convolution codec is used to realize the reorganization and structural coding of the abnormal characteristics of energy big data, and coupling-time capsule layer is used to capture the coupling-time characteristics of multi-energy. For thermal load, gas load and photovoltaic output, few researches focus on their energy characteristics, and a relatively complete energy characteristic index system is lacking. Therefore, according to the electrical load characteristic index and electricity-heat-gas-electricity, this section Common electrical load characteristics include load rate, minimum load rate and peak-valley ratio^15^. On this basis, the corresponding characteristic indexes of heat load, gas load, photoelectric output and coupling characteristic indexes of electricity-heat-gas-light time series are added to reflect the energy consumption characteristics of different multi-energy users in the integrated energy system. The expressions of electricity-heat-gas-light load rate and minimum load rate of multi-energy users in integrated energy system are:

4 $$ e_{R,j} = (\left| {y_{R,j} (n)} \right|^{2} - R_{2,R} ) \times y_{R,j} (n)^{ * } , $$

wherein, $y_{R,j} (n)$ is the load rate and minimum load rate of energy R, $R_{2,R}$ is the second-order statistical characteristic quantity of daily average load, and $y_{R,j} (n)^{ * }$ is the daily maximum load power. When analyzing the characteristics of electricity-heat-gas-light time series of multi-energy users in the integrated energy system with the feature extraction results as the input reference, the general method is to uniformly cluster the historical data of electricity-heat-gas-light time series of all multi-energy users to obtain the clustering results. However, direct global clustering has high complexity, long calculation time and unsatisfactory clustering results. Based on this, this chapter puts forward a hierarchical clustering framework for users of integrated energy system, combining the respective advantages of global clustering and local clustering to achieve the purpose of improving convergence speed and reducing calculation time, thus obtaining the multi-level index feature quantity as follows:

5 $$ x_{i} (n) = \sum\limits_{j = 1}^{M} {h_{ij} (n)^{T} } {\varvec{s}}_{j} (n) + v_{i} (n), $$

wherein, $v_{i} (n)$ is the physical resource utilization amount of information transmitted to the integrated energy system, ${\varvec{s}}_{j} (n)$ is the representation component of balanced clustering quality, and $h_{ij} (n)$ is the average distance between the clustering objects. The similarity between all samples in the data set is calculated respectively for quantitative evaluation, and the abnormal features of the information are detected to remove the interference information. The clustering results are evaluated according to the dispersion between classes and the compactness within classes, and the abnormal cluster detection output model of energy big data is obtained:

6 $$ AVG_{{\text{X}}} = \frac{1}{m \times n}\sum\limits_{x = 1}^{n} {\sum\limits_{y = 1}^{m} {\left| {G_{{\text{X}}} (x,y)} \right|} } , $$

where $G_{{\text{X}}} (x,y)$ is the corresponding intra-cluster error directivity function, and M and N are the vector and cluster center of clusters, respectively. In the energy data clustering channel, it is a private atomic broadcast channel, which is isolated from each other, and it is divided and managed by sorting service. The contents contained in the channel include the members of the organization belonging to the energy data channel, the chain codes deployed in the channel, the transaction information generated when trading in the channel, and the account book for storing these energy data information.

## Detection of abnormal clusters in big energy data

### Multi-energy data redundancy convolution coding and decoding

**Figure 1:**
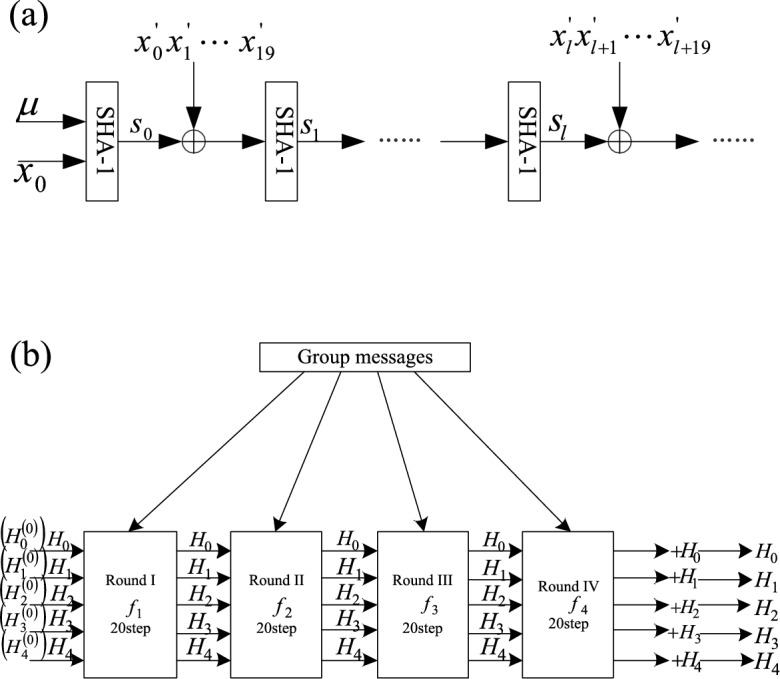
Redundant convolution codec.

A large number of cryptography techniques can be found in blockchain: the Merkle tree is built using hashing technique, the chain structure of blockchain is built based on hashing chain, the signature and verification of transactions in blockchain are realized using public key cryptography and hashing function, etc. Hashing function is a mathematical function, which can hash a message of any length in a limited and reasonable time range, and then get the output value, in which the length of the output value is fixed, and it is difficult to deduce the message backwards. However, large energy data usually have high dimensions and complex structures, and traditional anomaly detection methods may not be able to deal with these data effectively. The redundant convolutional encoder can reassemble and encode the abnormal features of the big energy data. By learning the local and global features of the data, it can better capture the abnormal patterns and abnormal clusters in the energy data. At the same time, the coupled time capsule layer is used to capture the coupled time characteristics of multiple energies to further improve the anomaly detection capability. The redundant convolutional encoder can extract more representative features by re-encoding the data, effectively reduce the data dimension, and integrate the time sequence information, so it is particularly suitable for anomaly clustering detection of large energy data. Therefore, redundant convolutional codec is used for data analysis, as shown in Fig. 1.

According to the redundant convolutional codec shown in Fig. 1, it is assumed that the rank value r and regularization parameter of plaintext factorized by hash algorithm converge to:

7 $$ \lambda_{i} = \mathop {\lim }\limits_{N \to \infty } \frac{1}{N}\sum\limits_{j = 0}^{N - 1} {\log \left( {{\varvec{R}}_{j} } \right)_{ii} } = 0, $$

wherein, $N$ is the number of data samples with arbitrary changes in the input plaintext, ${\varvec{R}}_{j}$ is the k-th coded plain code sequence, and $({\varvec{R}}_{j} )_{ii}$ means that the sender hashes the original electronic text to obtain a digital abstract matrix. With digital signature, the receiver can verify whether the received information is complete and judge whether the information has been tampered or lost in the transmission process. You can also judge whether the acquired data information is the original data according to the signature. The tensor model is constructed by correlation detection method, and the redundant convolution coding and decoding statistics of multi-energy data can be calculated as follows:

8 $$ W = \frac{{\overline{K} }}{\gamma } = \frac{1}{\gamma }\sum\limits_{k = 1}^{K} {\sum\limits_{n = 1}^{N} {kp_{k,n} } } , $$

wherein, $\gamma$ is the number of rules and protocols that have been set, $K$ is to minimize the similarity between classes, $k$ is to maximize the similarity within classes, and $p_{k,n}$ is a reward mechanism, assuming that each line of fi uniquely represents the ith entity. According to the above analysis, multi-energy users are continuously re-clustered until the DBI index of the clustering results no longer drops, and the optimal clustering results under the DBI index are obtained. In order to avoid incomplete evaluation of single cluster validity index, the calculation formulas of SSE index and contour coefficient are brought into adaptive k-means clustering algorithm, and the optimal clustering results under three indexes are obtained. Then, the three clustering results are cross-evaluated, and the final optimal clustering number and clustering results are obtained.

### Energy big data for abnormal cluster clustering analysis

The coupling time feature is synthesized by the fully connected linear regression layer to generate the component of the anomaly clustering feature, and then the energy time series data is converted into the characteristic value of the time series in the three-dimensional space. For the comprehensive energy system, the abnormal cluster analysis is carried out on the energy big data of massive multi-energy users, and the K adjacent sample values of abnormal data distribution are obtained as follows:

9 $$ P_{1J} = \sum\limits_{{d_{i} \in kNN}} {Sim(x,d_{i} )y\left( {d_{i} ,C_{j} } \right)} , $$

wherein, $x$ represents the amount of data collected and transmitted by energy big data of mass multi-energy users, $d_{i}$ represents the normal data component, $C_{j}$ represents the distribution matrix of energy big data sampling sensor nodes of mass multi-energy users, $Sim(x,d_{i} )$ represents the similarity, and $y(d_{i} ,C_{j} )$ represents the time domain sparse characteristic solution of energy big data of mass multi-energy users. The statistical characteristic quantity of abnormal cluster data of energy big data is constructed, and the conceptual function of abnormal cluster number distribution of energy big data is obtained as follows:

10 $$ d(t) = a(t)c(t) = \sum\limits_{n = 0}^{\infty } {d_{n} g_{c} \left( {t - nT{}_{c}} \right)} , $$

wherein

11 $$ d_{n} = \left\{ {\begin{array}{*{20}c} { + 1} \\ { - 1} \\ \end{array} \begin{array}{*{20}c} {} \\ {} \\ \end{array} \begin{array}{*{20}c} {a_{n} = c_{n} } \\ {a_{n} \ne c_{n} } \\ \end{array} \begin{array}{*{20}c} {} \\ {} \\ \end{array} } \right.(n - 1)T_{c} \le t \le nT_{c} , $$

wherein, $a(t)$ is the number of abnormal clusters at time t, $c(t)$ is the number of cluster centers at time t, $d_{n}$ is the distance threshold of the $n$-th data point in the cluster, $g_{c}$ is the global distribution of anomaly cluster centers, $nT{}_{c}$ is the time window size, $a_{n}$ is the exception score of the $n$-th data point in the cluster, and $c_{n}$ Is the cluster center of data point $n$.

Based on the linear layer, the characteristic diagram of electricity-heat-gas-light loads of multi-energy users is converted into one-dimensional form, and the abnormal cluster detection output of large energy data sets is as follows:

12 $$ Z_{n} = \sum\limits_{m = - \infty }^{\infty } {\left| {{\text{sgn}} [x(m)] - {\text{sgn}} [x(m - 1)]} \right|w(n)} , $$

wherein

13 $$ {\text{sgn}} [x] = \left\{ {\begin{array}{ll} {1}, & \quad {x \ge 0} \\ { - 1} & \quad {x < 0} \end{array} } \right. $$

14 $$w(n) = \left\{ {\begin{array}{*{20}ll}    {\frac{1}{{2N}}, \quad 0 \le n \le N - 1}  \\    {0, \quad {\text{ else}}}  \\   \end{array} } \right. $$

wherein, ${\text{sgn}} [x]$ is a symbolic function, $x(m)$ is the $m$ th data point, and $w(n)$ is the weight function.

Through sparse feature reorganization, the abnormal cluster detection model of energy big data of massive multi-energy users is constructed. Users can write specific code logic to meet the corresponding application programs, and then support the operation of related businesses. After the user chain code is deployed, it will run in a separate chain code container and communicate with the corresponding nodes through the chain code state machine. The client can use the chain code to access the account book. The implementation of its interface code can be written in Go language and other high-level programming languages. To install chain codes, you need to specify the installed channel, and multiple chain codes can be installed in a channel. When there are proper permissions between chain codes, they can call each other. To sum up, the abnormal cluster analysis of energy big data of massive multi-energy users is carried out, and the optimal number of multi-energy users is determined. The fully connected linear regression layer is used to integrate the coupling-time characteristics, and the double-layer iterative training method is used to adjust the model parameters and accelerate the convergence speed.

## Simulation and result analysis

Next, through the typical case of multi-energy users in the integrated energy system in northern China, the feasibility and effectiveness of the proposed multi-energy time series comprehensive prediction model is verified.. According to the analysis of cluster results, cluster result 1 has the largest number of multi-energy users. In order to keep the generality of data samples, the historical data of electricity-heat-gas-light time series of multi-energy users from cluster result 1 is selected as the input data sample to verify the feasibility of the proposed multi-energy comprehensive prediction model. Because of the different types of historical data of electro-thermal gas-optical time series, it is necessary to normalize them and convert the data to the same dimension, which is convenient for subsequent cluster analysis. The multi-energy user has a solar panel capacity of 10kW. The sampling interval of electric-thermo-gas-optical time series data is 1 h. Each month's data is divided into training data and test data. Taking the data of the whole year of 2020 as the research object, the data from January 1 to August 31 is the training data, and the data from September 1 to December 31 is the test data. The linear layer integrates the electricity-heat-gas-light time series data of multi-energy users into one-dimensional form; The regression layer generates the final prediction result of electricity-heat-gas-light time series of multi-energy users. In order to meet the accuracy and rapidity of multi-energy comprehensive prediction, the training times of the multi-energy comprehensive prediction model are set to 100 times, and the network parameters of the prediction model are randomly initialized and then updated. See Table 1 for the learning rate distribution.

**Table 1:** Parameters setting.

Neuron	Learning rate	Iterative steps
10	0.270	220
20	0.219	202
30	0.344	253
40	0.287	288
50	0.113	283
60	0.443	236
70	0.230	201
80	40.604	297

**Figure 2:**
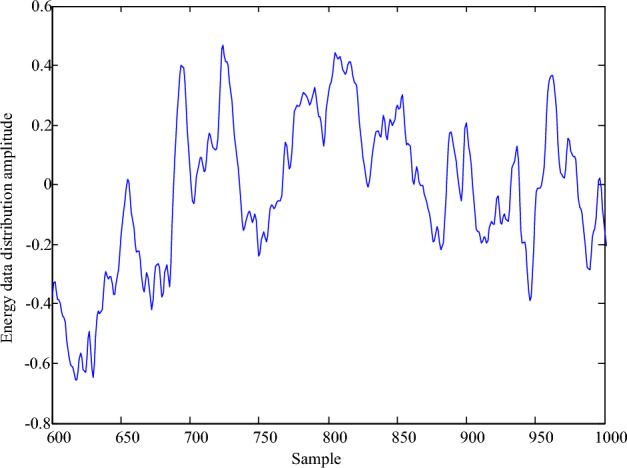
Time domain waveform of energy big data anomaly cluster identification.

The simulation test analysis shows that the number of characteristic sampling nodes of energy big data abnormal cluster is 240, the block area of energy big data cluster is 1000 m^2^, and the detection frequency of energy big data is 1200 kHz. The time domain waveform of data acquisition is shown in Fig. 2.

**Figure 3:**
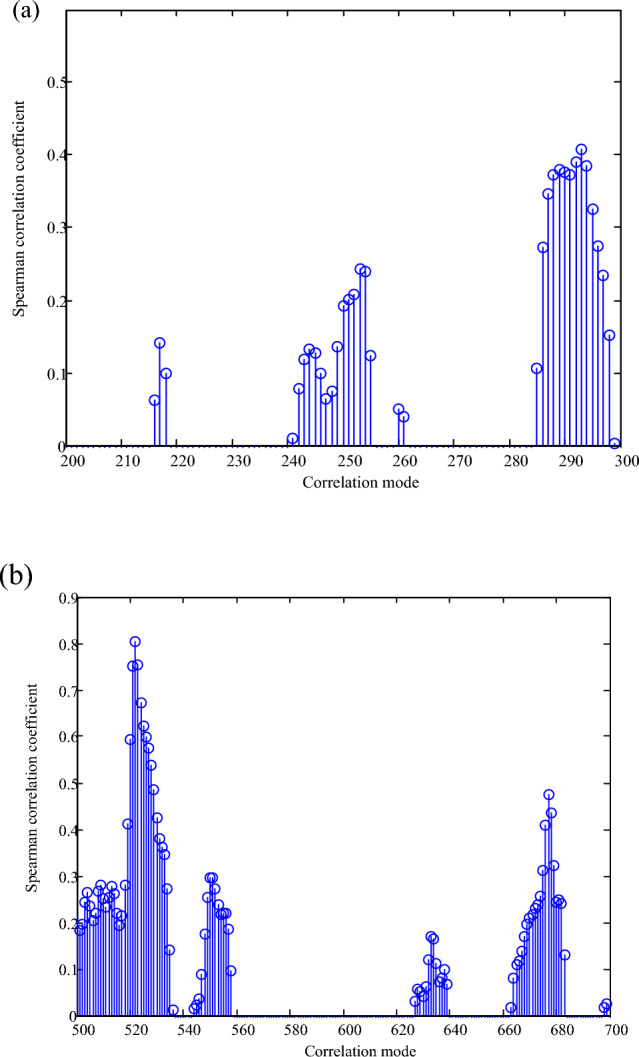
Spearman rank correlation coefficient of abnormal cluster distribution.

Taking the data of Fig. 2 as the research object, the abnormal cluster of energy big data is detected, and the Spearman rank correlation coefficient is obtained by encoding and decoding, as shown in Fig. 3.

**Figure 4:**
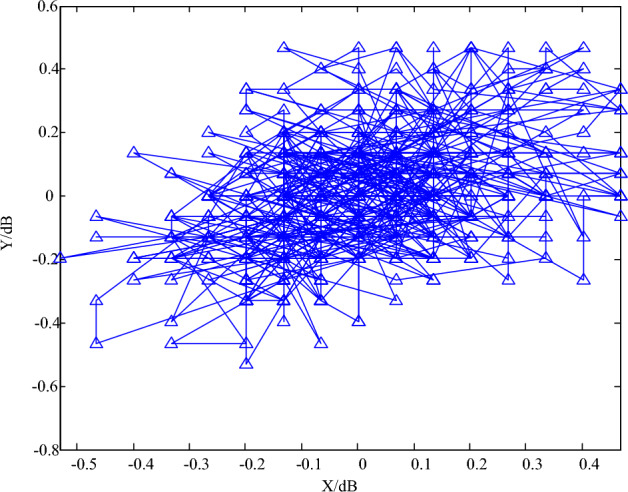
Abnormal cluster detection results of energy big data.

According to the analysis of Fig. 3, there is a certain correlation between multi-energy time series with users. For example, in correlation mode 1, Spearman rank correlation coefficients of electrical load, thermal load, gas load and photovoltaic output between users 1 and 2 are 0.8189, 0.7975, 0.6140 and 0.6023 respectively. It shows that there are strong temporal and spatial characteristics between the four loads of User 1 and User 2, and that User 1 and User 2 may be residential users and commercial users who are close to the commercial center, respectively, and the staff of residential users have more time to move in the commercial center. In addition, in the correlation model 11, Spearman rank correlation coefficients of electrical load, thermal load, gas load and photovoltaic output between user 1 and user 12 are 0.3041, 0.1923, 0.3088 and 0.3186, respectively. The correlation between the four loads of user 1 and the four loads of user 12 is weak, indicating that user 12 may be an industrial user far away from user 1, and there is less turnover and related activities between them. The analysis results of time–space characteristics between multi-user and multi-energy time series show the systematicness of multi-user and multi-energy time series in integrated energy system. Therefore, in order to improve the prediction accuracy of multi-user and multi-energy time series in integrated energy system, the time–space coupling characteristics among multi-users should be considered, and finally the abnormal cluster detection results are shown in Fig. 4.

As can be seen from Fig. 4, the feature clustering effect of anomaly detection of energy big data in this paper is good, and it has high performance in the detection and recognition of abnormal data. This shows that the method can effectively find abnormal patterns in energy data and cluster them, making the characteristics of abnormal data more obvious and easy to identify. This is because the proposed method can accurately determine the optimal number of multi-energy users, and use advanced linear layer technology to convert various feature maps of multi-energy users into one-dimensional forms, which not only simplifies the process of data processing, but also greatly improves the efficiency and accuracy of anomaly cluster analysis. Next, on the basis of the above tests, to further verify the applicability of the proposed method, literature^8^ method, literature^9^ method and literature^10^ method are taken as comparison methods, and detection accuracy, detection efficiency and detection error rate are selected as indicators to test the proposed method and the three comparison methods. Among them, the accuracy of abnormal clustering detection is tested, and the results are shown in Table 2. As can be seen from the results in Table 2, when the number of iterations reaches 500, the accuracy rate of anomaly detection of the proposed method can reach 98.7%, while the accuracy of anomaly detection of the method^8^, method^9^ and method^10^ are 89.1%, 86.0% and 89.9%, respectively. Compared with the results obtained by the four methods, it is concluded that the accuracy of anomaly cluster identification of energy big data in this paper is higher, indicating that the method in this paper has better anti-interference ability.

**Table 2:** Comparison of detection accuracy of abnormal clusters in energy big data.

Iterations	Detection accuracy/%			
	This method	Reference^4^	Reference^5^	Reference^6^
100	90.1	81.5	71.1	60.4
200	91.9	82.2	71.9	66.1
300	95.9	85.6	72.2	70.8
400	98.5	88.8	75.5	78.1
500	98.7	89.1	86.0	89.9

**Figure 5:**
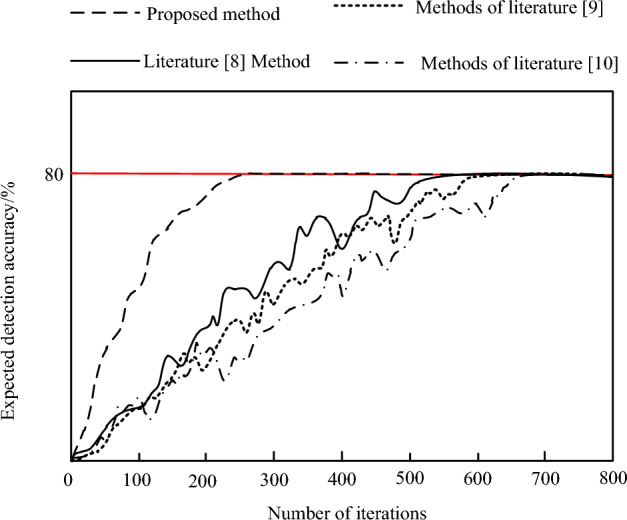
Comparison of convergence speed results.

Then, an expected detection is set, and the convergence speed of the four methods is statistically analyzed to analyze their detection efficiency. The results are shown in Fig. 5 below.

According to 5, the proposed method can achieve the expected detection accuracy when the number of iterations is 220; The three methods achieved the expected detection accuracy only when the number of iterations was 520, 600 and 695, respectively. Therefore, compared with the convergence speed of the four methods, it can be seen that the proposed method requires fewer iterations, indicating that it has faster detection efficiency and can quickly realize anomaly detection of energy big data.

**Figure 6:**
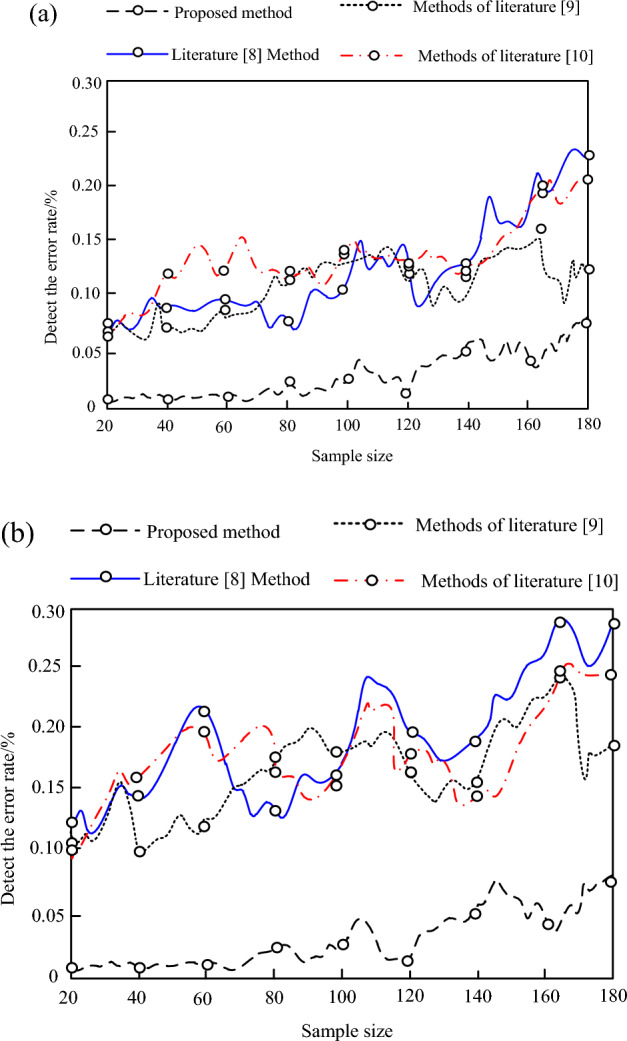
Test error rate results.

To further verify the reliability of the proposed method, 180 energy sample data were randomly selected from the training and testing sets, and the proposed method, literature^8^ method, literature^9^ method, and literature^10^ method were used to perform anomaly detection on the selected 180 energy sample data from the training and testing sets. The detection error rate is shown in Fig. 6.

According to Fig. 6, it can be seen that the proposed method has an anomaly detection error rate of less than 0.1 for both the training set energy sample data and the test set energy sample data, and when the data volume reaches 180, the results of the two datasets are consistent. The anomaly detection error rates of the methods in references^8–10^ for the energy sample data in the training set were 0.12, 0.23, and 0.20 respectively when the data volume reached 180. The anomaly detection error rates for the energy sample data in the test set were 0.17, 0.28, and 0.24, respectively. There are significant differences in the results of the three comparison methods between the training and testing sets. From this, comparing the detection results of the four methods, it can be seen that the detection error rate of the proposed method is lower than that of the three comparison methods, indicating that it can effectively complete the anomaly detection of energy big data and has good performance in the anomaly detection task of energy big data. The anomaly detection results on different datasets are relatively stable and will not fluctuate significantly due to changes in dataset size, which can effectively avoid overfitting problems and has reliability.

## Conclusions

To effectively solve the problems of high error rate and low detection efficiency caused by scattered abnormal cluster distribution and poor clustering in energy big data, a redundant convolutional encoder decoder based energy big data abnormal clustering detection method is proposed, providing a novel solution. This method enhances the accuracy and stability of anomaly detection by introducing redundant convolutional encoding, especially when dealing with scattered and poorly clustered anomaly clusters. And the energy consumption behavior characteristics of multi energy users were extracted, and the coupling time capsule layer and fully connected linear regression layer were used to deeply analyze and integrate the features. This ensures the comprehensiveness and accuracy of anomaly detection. In addition, by optimizing the determination of the number of multi energy users and the process of feature transformation, this method further improves the efficiency and accuracy of anomaly clustering detection in massive multi energy user energy big data. The simulation results show that the proposed method has excellent feature clustering performance, detection accuracy above 98.7%, fast convergence speed, and an error rate below 0.1, demonstrating superior performance. It can effectively provide an efficient and reliable anomaly detection method for the energy industry, which helps to improve energy utilization efficiency and management level.

## Data Availability

The data used to support the findings of this study are available from the corresponding author upon request.
